# Freezing-Induced Stress in mRNA-Lipid Nanoparticles During Lyophilization: Mechanistic Insights From Process and Formulation Studies

**DOI:** 10.1007/s11095-026-04039-x

**Published:** 2026-02-20

**Authors:** Anna Ruppl, Andrei Hutanu, Monika Köll-Weber, Andrea Allmendinger

**Affiliations:** 1 Department of Pharmaceutics, Institute of Pharmaceutical Sciences, University of Freiburg, Sonnenstr. 5, 79104 Freiburg I. Br., Germany; 2 ten23 Health AG, Mattenstr. 22, 4058 Basel, Switzerland

**Keywords:** Controlled nucleation, Freezing, Lipid nanoparticles, Lyophilization, mRNA

## Abstract

**Introduction:**

Lyophilization is a promising strategy to enhance the long-term stability of messenger RNA lipid nanoparticles (mRNA-LNPs). However, lyophilization-induced stresses can impact product quality and the underlying mechanisms remain poorly understood. In this study, we systematically investigated stresses that arise during the freezing step, during the initial stage of the lyophilization process.

**Methods:**

We examined the impact of different freezing protocols (freezing at 0.1, 0.5, and 1.5 K/min, plus controlled nucleation at -10°C) on mRNA-LNP stability. We also explored formulation strategies to mitigate freezing stress: (A) increasing mRNA-LNP concentration or adding empty LNPs to induce colloidal crowding, (B) adding Poloxamer 188 to reduce interfacial stress, (C) incorporating sucrose within LNPs to protect mRNA and reduce osmotic stress, and (D) adding NaCl or L-Methionine to modulate mRNA-lipid interactions. We evaluated particle size, polydispersity index, encapsulation efficiency (EE), mRNA integrity, and eGFP expression in HeLa cells.

**Results:**

Faster freezing minimized LNPS particle size increase by trend but reduced EE. Controlled nucleation improved EE but increased LNP particle size. However, eGFP expression was more influenced by particle size than EE.

**Conclusion:**

These findings provide a mechanistic understanding of how freezing-induced stresses affect mRNA–LNP quality. We hypothesize that cryo-concentration caused by slow freezing leads to increasing size of LNP particles, while higher ice-liquid interfacial stress caused by fast freezing reduces EE. As these effects follow opposing trends, optimizing freezing conditions is crucial. Understanding these mechanisms will guide rational formulation and lyophilization process design for mRNA-LNPs.

**Supplementary Information:**

The online version contains supplementary material available at 10.1007/s11095-026-04039-x.

## Introduction

Lyophilization has been established as a suitable approach to improve the long-term stability of messenger RNA lipid nanoparticles (mRNA-LNPs), eliminating the need for storage at frozen temperatures [1–3].

Although the final dry state of the lyophilizate stabilizes mRNA-LNPs, the lyophilization process itself including both freezing and drying processes generates various stress conditions and may impair product quality. While lyophilization typically increases particle size, it also tends to reduce encapsulation efficiency (EE) [3–6]. On the other hand, it has been recently demonstrated by Fan and colleagues that specially changes in mRNA encapsulation and mRNA stability, two cargo-related attributes, more significantly influence cellular translation efficiency than particle size changes. In detail, Fan *et al*. conducted a lyoprotectant and buffer screening to investigate the physicochemical and structural properties of lyophilized eGFP-mRNA-LNPs. Most formulations exhibited significant increased particle size, reduced EE, and changes in morphology [7]. However, it remains unclear, which specific stresses during the lyophilization process are responsible for these changes in particle characteristics.

To protect both the LNP structure and the mRNA cargo, formulation excipients such as cryo- and lyoprotectants need to be carefully selected [6, 8, 9].

In 1993, Crowe *et al*. proposed the water replacement hypothesis, explaining how disaccharides like trehalose protect liposomes during drying. These sugars form hydrogen bonds with phospholipid head groups in place of water, maintaining the bilayer in a liquid crystalline state during drying and preventing phase transitions upon rehydration, thereby preserving membrane structure [10]. Another explanation for stabilization during freezing is the vitrification theory, which suggests that particles are immobilized in an amorphous, highly viscous matrix with reduced molecular mobility, thereby limiting fusion and aggregation [11, 12]. However, the precise mechanism by which lyophilization stabilizes mRNA-LNPs remains currently unclear.

Several stress factors may affect mRNA-LNPs during lyophilization, particularly during the freezing step. First, the formation of ice and excipient crystals may damage liposomes. The presence of large crystals has been associated with leakage [13]. Moreover, the size and number of ice crystals determine the interfacial area. Stress at ice-liquid interfaces may lead to adsorption or structural damage of colloidal systems [14], as previously described for proteins [15]. Freezing also induces cryo-concentration. As temperature decreases and ice crystals form, only pure water is incorporated into the hexagonal ice lattice. Solutes, such as sugars and buffer components, and nanoparticles are excluded and concentrate in the remaining unfrozen liquid. This fraction becomes increasingly concentrated until it solidifies upon reaching the eutectic temperature or the glass transition temperature of the maximally freeze-concentrated solution. Cryo-concentration may increase the ionic strength and the osmotic pressure of the maximum freeze concentrate compared to the initial formulation. This effect is especially pronounced under slow cooling rates, where larger ice crystals may form [16]. Additional formulation-dependent stress factors may include shifts in pH and liquid–liquid phase separation. pH changes may occur if one buffer component crystallizes earlier than another, as in sodium phosphate buffer systems [17]. Liquid–liquid phase separation, observed in certain polymer mixtures such as PEG and dextran [18], is characterized by the presence of two distinct glass transition temperatures [14, 19].

Recent publications about lyophilization of mRNA-LNPs have focused primarily on optimizing formulation composition by investigating various excipients to improve overall stability [5–8, 20]. In contrast, only a few studies have addressed the optimization of lyophilization process parameters. A patent by Moderna, Inc. reported that a low residual moisture enhances their long-term stability. Although residual moisture below 0.5% was achieved using a secondary drying temperature of 40 °C, this condition led to reduced mRNA integrity. Unexpectedly, incorporating an annealing step prior to drying facilitated harsher secondary drying conditions without compromising mRNA integrity, likely due to the formation of larger ice crystals [21]. To date, two publications have compared different freezing condition for mRNA-LNPs. Meulewaeter *et al*. applied spin-freezing using both slow and fast cooling rates. Their supplementary data showed that a fast cooling rate preserved the enhanced green fluorescent protein (eGFP) mRNA-LNP better with higher eGFP-expression and better maintained polydispersity index (PDI) [5]. In another publication, Wang *et al*. compared freezing methods during lyophilization for monkeypox (mpox) mRNA-LNPs including liquid nitrogen, controlled nucleation (CoN) by vacuum-induced surface freezing, and conventional freezing at rates of 1 K/min and 0.45 K/min. Cooling with liquid nitrogen and CoN resulted in fragmented cakes. Additionally, cooling with liquid nitrogen caused a strong increase in size and a decrease in EE, both with high variability. Best results were obtained using conventional freezing. The slower cooling rate of 0.45 K/min yielded the smallest particle size and the highest EE after lyophilization of mpox mRNA-LNPs. Accordingly, a cooling rate of 0.45 K/min was employed in their subsequent experiments These experiments involved the investigation of additional process parameters and the execution of a long-term stability study at 4 °C [3].

Despite these findings, there is still a lack of systematic understanding and optimization of the lyophilization process for mRNA-LNPs. The specific impact of the freezing step has not been thoroughly investigated, and many studies omit detailed process parameters, especially those related to freezing. In particular, the interaction between stress conditions during freezing and stabilizing formulation excipients requires further investigation. Our aim was to gain a mechanistic understanding of how freezing-induced stresses affect mRNA–LNP characteristics, thereby providing insights to support process design and targeted formulation development.

The present study investigated the impact of different freezing conditions during lyophilization on the stability of eGFP-mRNA-LNPs. We compared freezing at rates of 0.1, 0.5, and 1.5 K/min with a cycle utilizing CoN at −10°C. For these experiments, we used an established formulation for mRNA-LNPs, which contained 10% (w/v) sucrose in Tris buffer [5, 6, 22, 23]. In addition, we explored various formulation strategies to enhance mRNA-LNP stability during the freezing process. These included: (A) increasing mRNA-LNP concentration or adding empty LNPs to improve stability via colloidal crowding, (B) adding Poloxamer 188 as a surfactant to reduce interfacial stress, (C) incorporating sucrose within the LNPs to protect mRNA and reduce osmotic pressure, (D) adding NaCl or L-Methionine to the aqueous phase to influence interactions between mRNA and ionizable lipid. To assess stability, we evaluated size, PDI, EE, mRNA integrity, and eGFP-expression in HeLa cells.

## Materials and Methods

### Preparation of LNPs

LNPs were prepared according to a protocol published recently by Ruppl *et al*. [6]. In brief, LNPs were prepared by T-mixing of the organic phase containing SM-102 (DC Chemicals, Shanghai, China), cholesterol (Sigma-Aldrich, St. Louis, USA), DSPC (Lipoid, Ludwigshafen, Germany), and DMG-PEG2000 (Avanti Polar Lipids, Birmingham, USA) in a molar ratio of 50: 38.5: 10: 1.5, and the aqueous phase containing CleanCap™ EGFP-mRNA (TriLink, San Diego, USA) or polyA (ABP Biosciences, Virginia, USA) dissolved in 50 mM citrate buffer pH 4.0. After mixing, LNPs were dialyzed into 20 mM Tris buffer pH 7.4. Formulations are summarized in Table I. Excipients were added either to the aqueous phase (protectant/excipient inside) or after dialysis (protectant/excipients outside). Kolliphor P 188 (Poloxamer 188) was from BASF, L-Methionine and sucrose from Pfanstiehl, and NaCl from Roth. Formulations were filtered through 0.20 μm PVDF filters (Chromafil, Faust, Klettgau, Germany), and 300 μL aliquots were filled into clear 2 mL vials (Schott, Müllheim, Germany) and partly closed with lyo stoppers (Datwyler Pharma Packaging, Alken, Belgium) prior lyophilization.

**Table I:** Formulation Compositions used to Investigate Freezing-Conditions. *Also Referred to as Standard Formulation

Formu-lation No	Abbreviation	Cargo	c_eGFP-mRNA_ (μg/mL)	Protectant (% w/v) inside	Protectant (% w/v) outside	Additional excipient inside	Additional excipient outside
1	mRNA*	eGFP-mRNA	20	-	10% sucrose	-	-
2	mRNA Suc	eGFP-mRNA	20	10% sucrose	10% sucrose	-	-
3	polyA	polyA	20	-	10% sucrose	-	-
4	high conc	eGFP-mRNA	100	-	10% sucrose	-	-
5	+ empty LNPs	eGFP-mRNA	20	-	10% sucrose	-	empty LNPs
6	Poloxamer	eGFP-mRNA	20	-	10% sucrose	-	0.05% Poloxamer 188
7	NaCl inside	eGFP-mRNA	20	-	10% sucrose	50 mM NaCl	-
8	L-Met inside	eGFP-mRNA	20	-	10% sucrose	50 mM L-Methionine	-

To prepare formulation 4 (high concentrated mRNA-LNPs), LNPs were concentrated after dialysis using Amicon® Ultra-4 Centrifugal Filter Devices (Merck, Darmstadt, Germany) at 4 °C and 3000 g. The same procedure was applied to empty LNPs, which were prepared by mixing citrate buffer with the lipid-containing organic phase.

To confirm the lipid concentrations of both mRNA-LNPs and empty-LNPs, a reversed-phase high-performance liquid chromatography method with charged-aerosol-detector, adapted from Bender *et al*. [24] and modified with a reduced flow rate of 1.8 mL/min, was used (data not shown). For formulation 5 (mRNA-LNPs + empty LNPs), empty LNPs were added until the total SM-102 concentration matched the one of formulation 4 (high concentrated mRNA-LNPs).

### Lyophilization

Lyophilization was performed on a pilot freeze-dryer Epsilon 2-6D (Christ, Osterode, Germany) equipped with a LyoCoN unit. Filled vials were placed onto the middle shelf and surrounded with placebo vials containing 10% sucrose in 20 mM Tris buffer. Vials were frozen to −45°C. Three cooling rates were examined: 0.1 K/min, 0.5 K/min, and 1.5 K/min. In an additional cycle, controlled nucleation was applied using an ice fog technique. To this end, the samples were equilibrated for 15 min at a shelf temperature of −10°C. Subsequently, nucleation was induced at a vacuum of 10 mbar, with the shelf temperature decreased at a rate of 0.5 K/min before and after.

Shelves were held at −45°C for one hour prior primary drying, which was performed at 0.13 mbar and −20°C. Comparative pressure measurement (< 0.1% difference between Pirani and capacitance gauge) was used to determine the end of primary drying before increasing the temperature at a rate of 0.2 K/min to 25 °C for 5 h for secondary drying. After release of the vacuum, vials were stoppered at 750 mbar.

Lyophilized samples were reconstituted in 275 μL RNase-free water before analysis. Three vials per formulation and freezing condition were analyzed unless otherwise stated. Three vials from the same lyophilization cycle were analyzed as triplicate.

### Dynamic Light Scattering

Dynamic light scattering was used to determine size and PDI of LNPs. Measurements were performed using a Zetasizer Nano ZS (Malvern Panalytical, Kassel, Germany). Samples were diluted 1:8 in 20 mM Tris buffer containing 10% (w/v) sucrose and equilibrated for 120 s at 25°C. Detection occurred at 173° backscattering with automatic attenuation and triplicates were performed consisting of 10 runs of 10 s.

### RiboGreen Assay

Encapsulation efficiency (EE) of LNPs was determined in technical duplicates using Quant-iT RiboGreen Assay (ThermoFisher) and black 96-well plates (Greiner bio one, Frickenhausen, Germany) as described by Ruppl *et al*. [25]. Briefly, LNPs were diluted using either TE buffer or 2% (v/v) Ecosurf solution. Subsequently, 100 μL of RiboGreen reagent, diluted 1:400, was added to each well. Following a 5-min incubation protected from light, fluorescence was measured using a microplate reader (FLx800, BioTek, Vermont, USA) with an excitation wavelength of 485 nm and an emission wavelength of 528 nm. EE was determined by calculating the proportion of encapsulated nucleic acid relative to the total nucleic acid content.

### Capillary Gel Electrophoresis (CGE)

Analysis was carried out using a SCIEX PA800 Plus system (Brea; USA) equipped with a solid-state laser with an excitation wavelength of 488 nm and a 520 nm bandpass emission filter (Cat. no. 65–699) from Edmund Optics (Barrington; USA), a 30 kV power supply and a temperature-controlled autosampler (±  2 °C). LNP samples were purified following the instructions from the Gene Jet RNA purification kit (Thermo K0731). Before injection, the sample was heated at 70 °C for 2 min followed by 5 min in ice. The separation gel buffer consisted of 2% PVP, 4 M Urea in 1xTBE solution with 1:25,000 diluted SYBR Green II. A bare fused silica capillary with a 50 μm internal diameter and 30 cm effective length was used for the separation (Cat. No. TSP-050375, Polymicro Technologies/Molex LLC). The separation voltage was 6 kV using reverse polarity. The samples were injected by applying –4 kV during 2–6 s. Capillary temperature was set to 25 °C and 10 °C was used for the autosampler. Data were acquired and analyzed using Empower software.

### Cell Culture and Flow Cytometry

HeLa cells were cultured and seeded according to the protocol described by Ruppl *et al*. [25]. In brief, 60,000 cells/well were seeded in 24-well plates with 1 mL of culture medium. After 24 h, a defined volume of LNPs was added to achieve a final concentration of 0.5 μg/mL encapsulated mRNA, based on the liquid formulation before lyophilization. The applied volume was not adjusted for variations in EE. Untreated cells served as a negative control, while Lipofectamine 2000 (Thermo Fisher Scientific) was used as a positive control. Following a 24-h incubation, cells were analyzed via flow cytometry to evaluate mRNA-mediated eGFP expression in HeLa cells.

Measurements were performed in technical triplicates using a BD LSRFortessa system (Heidelberg, Germany). A total of 10,000 events within the “live cell gate” (SSC-A/FSC-A) were recorded, followed by doublet exclusion using FSC-H/FSC-A. Data were reported as the median fluorescence intensity (MFI) of the single-cell population.

### Physico-Chemical Properties

pH and osmolality were measured using an InLab Micro electrode (Mettler Toledo) and a semi-micro osmometer K 7400 (Knauer, Berlin, Germany).

### Karl-Fischer Titration

Residual moisture content of the lyophilizates was quantified in triplicate using volumetric Karl Fischer titration on a 915 KF Ti-Touch instrument (Metrohm, Herisau, Switzerland). Samples were dissolved in anhydrous methanol (Merck, Darmstadt, Germany). The methanol suspension was drawn up with a syringe and injected into the titration vessel containing Aquastar® solvent (Merck). Titration was carried out using Hydranal Titrant 2 (Honeywell, Seelze, Germany).

### Cake Appearance

Cake appearance was assessed by visual inspection, and representative images were captured against a dark background.

### Statistical Analysis

All data are expressed as mean ± standard deviation. Statistical analyses were performed using GraphPad Prism 10. Differences between groups (e.g. freezing conditions, formulations, liquid/lyophilized) were analyzed using a two-way ANOVA with Tukey’s multiple comparisons test. The following terminology was used: * *p* < 0.05; ** *p* < 0.01; ***p < 0.001; ****p < 0.0001; n.s. not significant *p* > 0.05.

## Results

### Impact of Freezing-Rate and Controlled Nucleation

To investigate the influence of the freezing step during lyophilization of LNPs, we tested eGFP-mRNA- and polyA-LNPs using freezing protocols at 0.1 K/min, 0.5 K/min, and 1.5 K/min, respectively. A fourth cycle employed controlled nucleation induced by an ice fog technique at a nucleation temperature of –10°C. All LNPs were formulated with 10% (w/v) sucrose in 20 mM Tris buffer pH 7.4. The first formulation contained eGFP-mRNA (labeled “mRNA” in graphs). The second formulation contained eGFP-mRNA but included an additional 10% (w/v) sucrose added to the aqueous phase prior to the mixing step (labeled “mRNA Suc” in graphs). The third formulation used polyA as a surrogate for mRNA, a common approach during technical development.

The nucleation temperatures in the cycles without CoN ranged from −  19 °C to −  13 °C across all cooling rates. Freezing times were reported for the formulations of 9 ± 3 min at 0.1 K/min, 4 ± 1 min at 0.5 K/min, and 3 ± 0 min at 1.5 K/min.

A similar trend was observed for stress time, the interval from nucleation until reaching the glass transition temperature (T_g_') of the maximally freeze-concentrated solution (–32°C for sucrose). This was approximately 2–3 h at 0.1 K/min, 26 ± 4 min at 0.5 K/min, and 10 ± 2 min at 1.5 K/min.

For CoN, freezing time was 11 min and stress time 56 min.

We evaluated the cake appearance and residual moisture of the lyophilizates. Samples prepared with the different cooling rates produced intact cakes with little shrinkage, while lyophilizates from the CoN cycle showed homogenous cake appearance without any defects (Tab. S-1). The use of CoN resulted in lower residual moisture compared to conventional freezing (Fig. S-1).

**Fig. 1:**
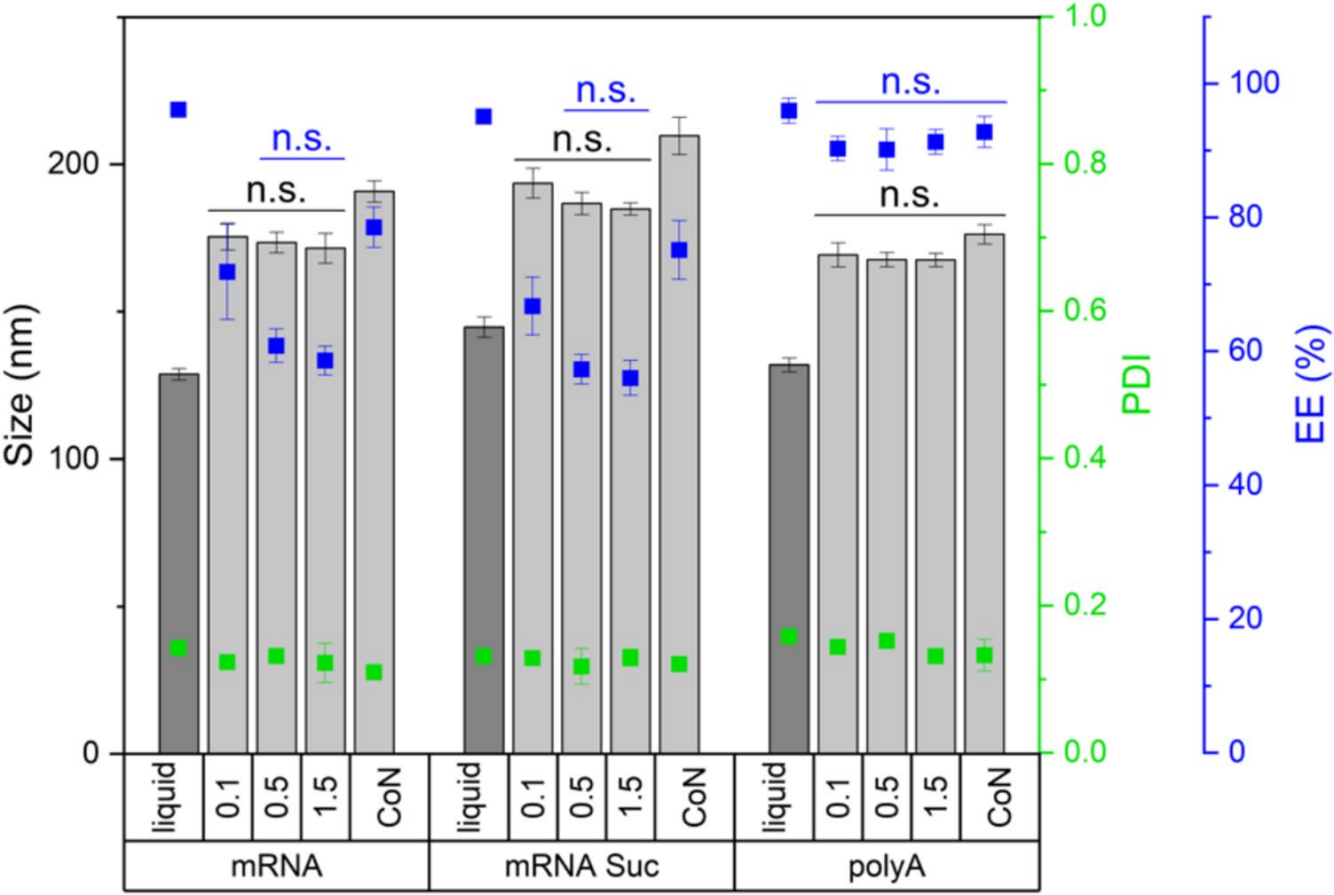
LNP characteristics (size, PDI, and EE) of the liquid formulation and after lyophilization with freezing protocols at 0.1 K/min, 0.5 K/min, or 1.5 K/min or using controlled nucleation (CoN) comparing eGFP-mRNA-LNPs formulated with 10% sucrose outside (mRNA), eGFP-mRNA-LNPs formulated with 10% sucrose inside and outside (mRNA Suc), and polyA-LNPs formulated with 10% sucrose outside (polyA). Statistical analysis was performed separately for size, PDI, and EE using a two-way ANOVA with Tukey’s multiple comparisons test. The measurements were executed in triplicate, with three vials being analyzed for each lyophilization cycle. n.s. not significant *p* > 0.05.

We measured particle size, PDI, and EE in the liquid formulation and after reconstitution of lyophilized samples with RNase-free water (Fig. 1).

The LNP formulations showed an increase in particle size following lyophilization. During conventional freezing, both eGFP mRNA formulations exhibited an average increase of approximately 50 nm, while the polyA-LNPs showed an increase of about 35 nm. Differences between cooling rates were not statistically significant for any formulations but a trend for slightly stronger increase in size with slower freezing was visible. However, inducing ice nucleation (CoN) at −10°C led to a more pronounced size increase in both eGFP-mRNA-containing formulations, while the size increase in polyA-LNPs remained comparable. The PDI remained stable regardless of lyophilization condition.

EE values in the liquid state were similar across all formulations. However, following lyophilization, EE decreased, with a stronger decline observed for the eGFP mRNA-containing LNPs compared to those with polyA, where changes were only little and not significant between freezing conditions. The most substantial EE losses occurred with increasing cooling rates to 0.5 K/min and 1.5 K/min. Conversely, the slowest rate (0.1 K/min) preserved EE better, and the highest EE for mRNA-LNPs after lyophilization was observed with CoN.

**Fig. 2:**
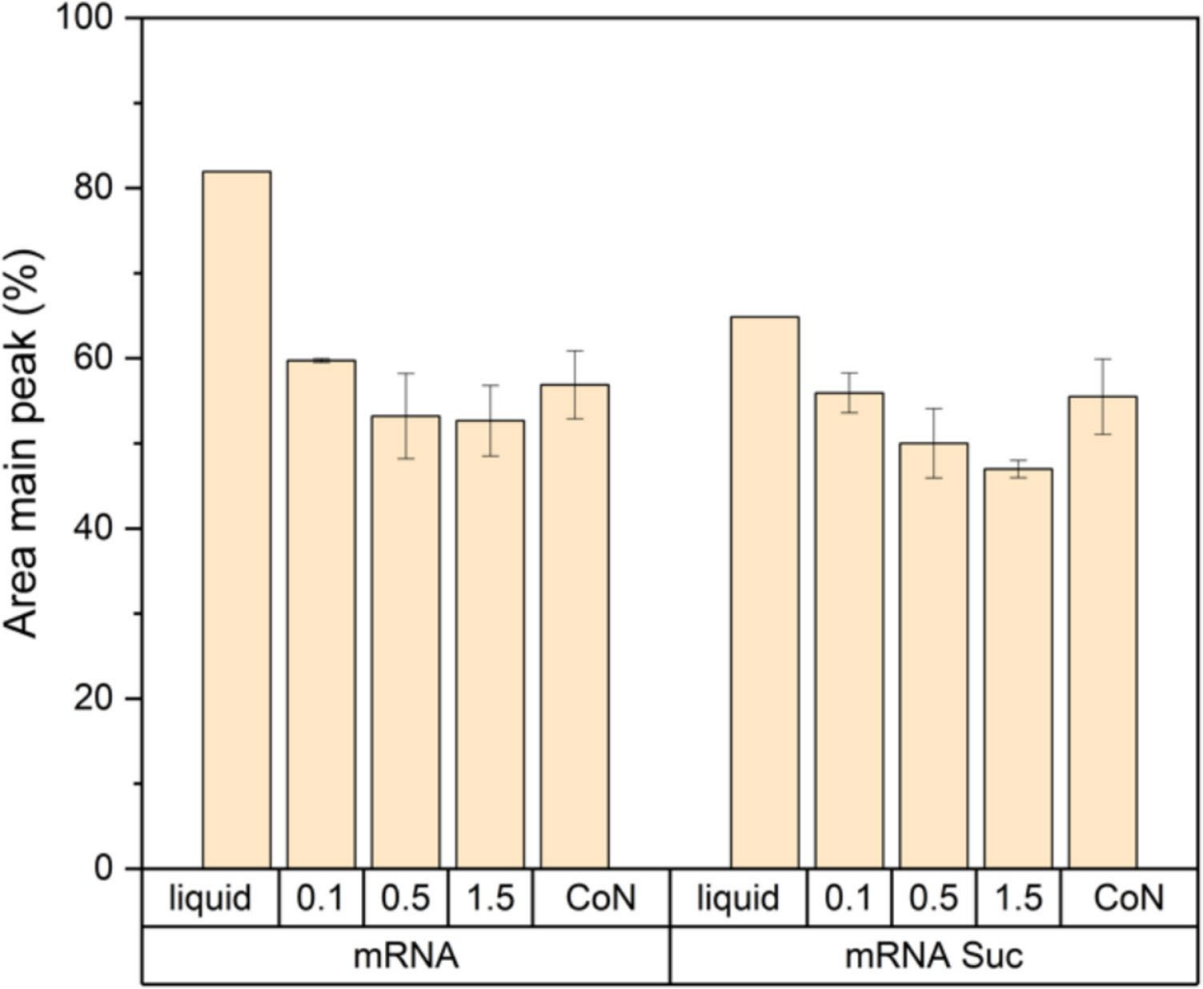
mRNA integrity determined by capillary electrophoresis of the liquid formulation and after lyophilization with freezing protocols of 0.1 K/min, 0.5 K/min, or 1.5 K/min or using controlled nucleation (CoN) for eGFP-mRNA-LNPs formulated with 10% sucrose outside (mRNA) and eGFP-mRNA-LNPs formulated with 10% sucrose inside and outside (mRNA Suc). Statistical analysis was performed using a two-way ANOVA showing that there are no significant variations between freezing-conditions or addition of sucrose inside the LNPs. The measurements for the lyophilized samples were executed in triplicate, with three vials being analyzed for each lyophilization cycle. The liquid samples are only a single measurement

Next, we assessed mRNA integrity using CGE (Fig. 2). Although mRNA integrity declined after lyophilization, statistical analysis via two-way ANOVA revealed no significant differences among the freezing conditions.

**Fig. 3:**
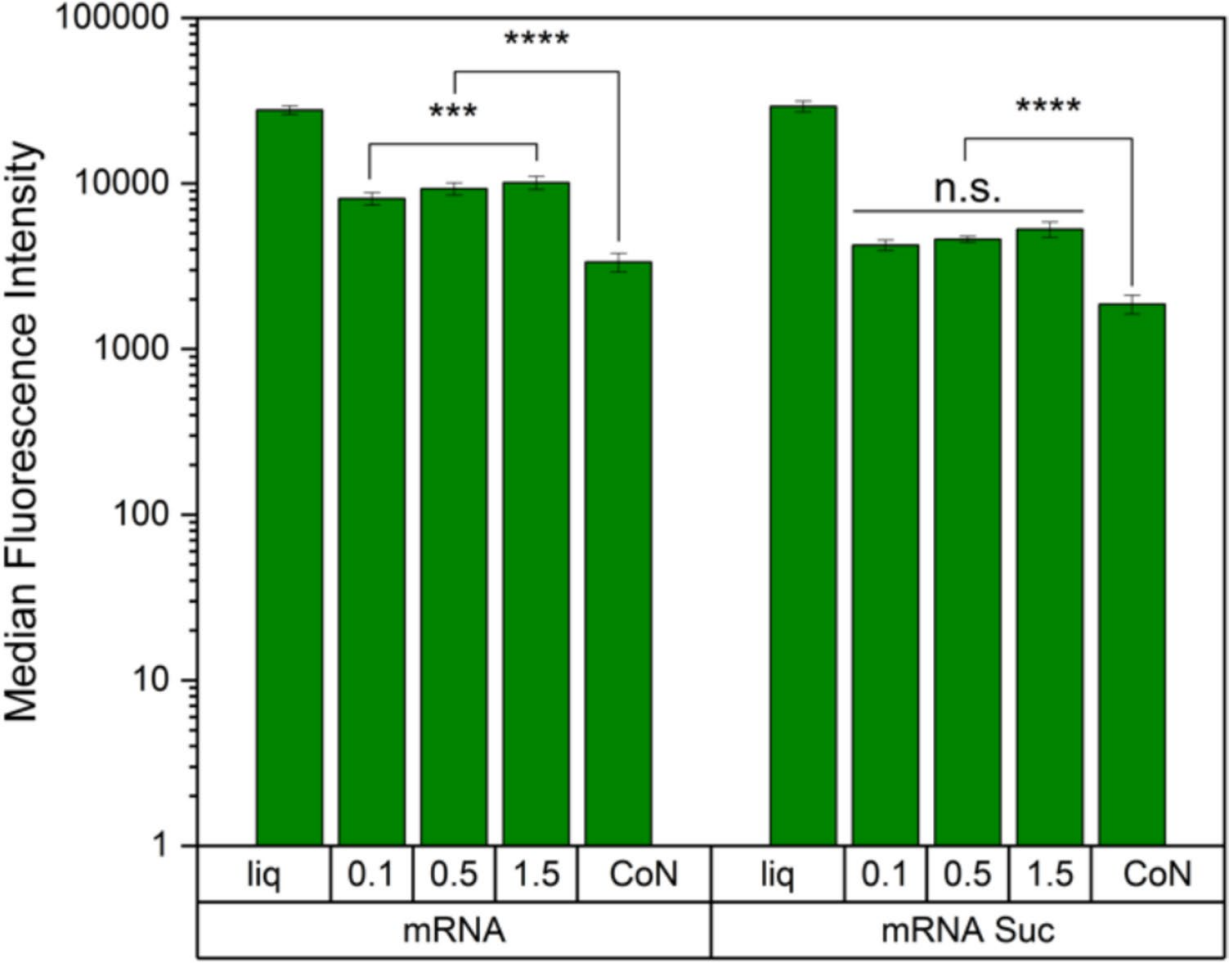
eGFP-expression in HeLa cells reported as median fluorescence intensity. eGFP-mRNA-LNPs formulated with 10% sucrose outside (mRNA) and eGFP-mRNA-LNPs formulated with 10% sucrose inside and outside (mRNA Suc) were investigated as liquid formulation (liq) compared to the reconstituted samples after lyophilization using freezing protocols of 0.1 K/min, 0.5 K/min, or 1.5 K/min or using controlled nucleation (CoN). Statistical analysis was performed using a two-way ANOVA with Tukey’s multiple comparisons test. The measurements were executed in triplicate, with three vials being analyzed for each lyophilization cycle. ***p < 0.001; ****p < 0.0001; n.s. not significant *p* > 0.05.

To evaluate the impact of freezing-conditions *in vitro*, we measured eGFP expression in HeLa cells using flow cytometry (Fig. 3). The same LNP volume was applied across samples without correcting for EE. While MFI decreased after lyophilization for all freezing conditions and formulations, the mRNA formulation without internal sucrose and a freezing protocol at 0.1 K/min resulted in significantly lower MFI compared to 1.5 K/min. In contrast, when sucrose was added prior to mixing, no significant differences in MFI were observed among the cooling rates. For both formulations, CoN resulted in significantly lower MFI than conventional freezing.

In summary, PDI was not affected by lyophilization, while particle size increased, particularly in samples processed with CoN. The cooling rate had no significant effect on size, and mRNA integrity was not influenced by the freezing condition. The most notable differences were observed in EE, which was best preserved with CoN and decreased most with conventional freezing at 0.5 K/min and 1.5 K/min as studied for eGFP mRNA formulations. Interestingly, the differences in EE were not reflected in eGFP expression. Rather, MFI appeared to be inversely correlated with particle size, meaning the smaller the particle size, the higher the MFI.

### Formulation Strategies

**Fig. 4:**
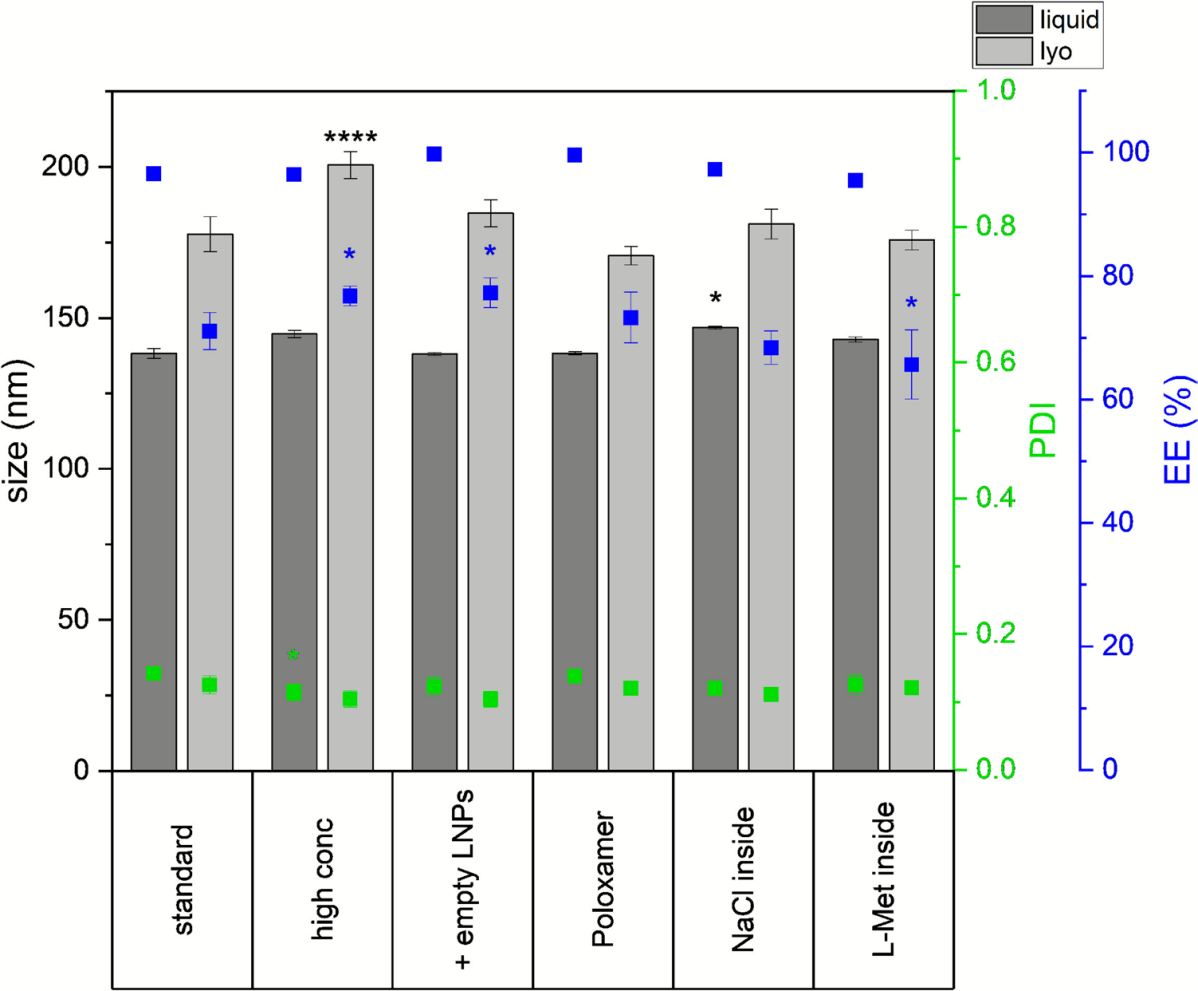
LNP characteristics (size, PDI, and EE) before (liquid) and after (lyo) lyophilization. LNPs were generally formulated with 10% (w/v) sucrose and 20 μg/mL mRNA (referred to as “standard” formulation). Additionally, the following formulations were tested: 100 µg/mL mRNA (high conc), addition of empty LNPs to a liquid content equivalent to the high conc formulation (+ empty LNPs), 0.05% Poloxamer 188 (Poloxamer), 50 mM NaCl were added to the aqueous phase before the mixing step (NaCl inside), and 50 mM L-Methionine were added to the aqueous phase before the mixing step (L-Met inside). Statistical analysis was performed separately for size, PDI, and EE using a two-way ANOVA with Tukey’s multiple comparisons test and significant differences compared to the standard formulation are shown. The measurements were executed in triplicate, with three vials being analyzed for each formulation. * *p* < 0.05; ****p < 0.0001; not significant *p* > 0.05.

**Fig. 5:**
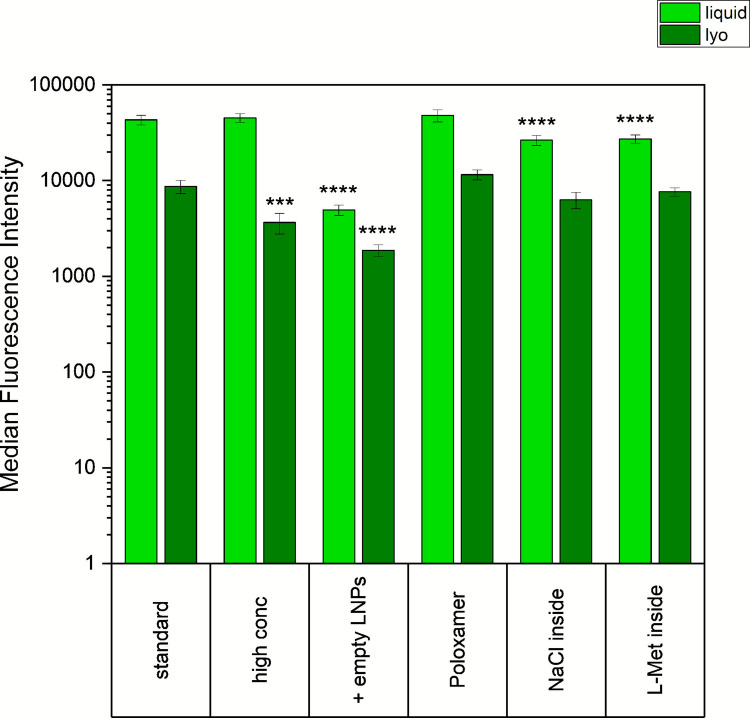
eGFP-expression in HeLa cells reported as median fluorescence intensity. LNPs were generally formulated with 10% (w/v) sucrose and 20 ug/mL mRNA (referred to as “standard” formulation). Additionally, the following formulations were tested: 100 µg/mL mRNA (high conc), addition of empty LNPs to a liquid content equivalent to the high conc formulation (+ empty LNPs), 0.05% Poloxamer 188 (Poloxamer), 50 mM NaCl were added to the aqueous phase before the mixing step (NaCl inside), and 50 mM L-Methionine were added to the aqueous phase before the mixing step (L-Met inside). Statistical analysis was performed using a two-way ANOVA with Tukey’s multiple comparisons test. Significant differences within liquid or lyophilized state compared to the standard formulation are shown. The measurements were executed in triplicate, with three vials being analyzed for each formulation. ***p < 0.001; ****p < 0.0001; not significant *p* > 0.05.

Based on the results described above, we proceeded with further experiments using eGFP-mRNA-LNPs using a freezing protocol at 1.5 K/min, as this produced the strongest drop in EE. Various formulation strategies were tested alongside our standard formulation, which contained 10% (w/v) sucrose and 20 µg/mL mRNA (Fig. 4). No difference in pH (6.9–7.2) and osmolality (366–390 mOsmol/kg) was measured for the tested formulations.

(A) Colloidal Crowding
  The common assumption is that dilution increases the colloidal stability of particles because the likelihood of collision is reduced. Davari and colleagues have recently presented that the addition of empty LNPs mitigates the negative effects of freeze/thaw cycles and lyophilization on mRNA-LNPs by colloidal crowding [26]. When nanoparticles are crowded together, their surfaces are more likely to be surrounded by similar particles, reducing the exposure of individual particle surfaces.
  To investigate colloidal crowding for LNPs, we examined the impact of eGFP mRNA concentration as well as LNP concentration. A high-(mRNA)-concentration formulation was prepared with a five-fold increase in both mRNA and lipid content (labelled “high conc” in graphs), and another formulation was prepared with a five-fold increase in lipid concentration, achieved by adding empty LNPs while keeping mRNA concentration constant (labelled “ + empty LNPs” in graphs). Initial LNP characteristics were comparable across the three formulations, with the high-mRNA-concentration formulation showing a slightly lower PDI (Fig. 4). Following lyophilization, the high-mRNA-concentration formulation exhibited a significantly higher increase in particle size, whereas the formulation with the addition of empty LNPs did not. Both formulations with higher lipid concentration preserved EE better than the standard formulation (Fig. 4). mRNA integrity was not impacted by the elevated concentrations of mRNA-LNPs or addition of empty LNPs (data not shown). eGFP expression levels in the liquid state were similar between the standard and high-mRNA-concentration formulation. However, the formulation containing empty LNPs showed a substantial decline in eGFP expression and reduced cell viability (data not shown). After lyophilization, eGFP expression declined significantly in all three formulations. The lowest eGFP expression was observed in the formulation with empty LNPs, while the highest was retained in the standard formulation (Fig. 5).
(B) Interfacial Protection
  Next, we investigated whether the addition of a surfactant could mitigate interfacial stress during lyophilization. Only few surfactants are approved and commonly used for parenteral application: Polysorbate 20, Polysorbate 80, Poloxamer 188, and Polyoxyl-15-hydroxystearate [27].
  However, polysorbates have the drawback of being susceptible to hydrolytic and oxidative degradation due to their ester bonds and unsaturated fatty acids [28–30]. Poloxamer has emerged as an alternative surfactant due to lack of ester bonds [31]. It has previously shown protective effects for proteins, extracellular vesicles, and viruses by reducing adsorption at ice-liquid interfaces [32–34]. Although constraints have been reported for protein drug products when combined with silicon oil [35], we chose to investigate the effects of Poloxamer 188 due to these protective effects.
  In our experiments, the addition of 0.05% Poloxamer 188 had no significant effect on size, PDI, EE, mRNA integrity (data not shown), or eGFP expression, either before or after lyophilization, compared to the standard formulation.
(C) Protect mRNA and Reduce Osmotic Pressure
  We further investigated whether adding sucrose to the aqueous phase prior to the mixing step would positively impact mRNA integrity or overall LNP characteristics, including EE, by protecting the mRNA or reducing osmotic pressure. mRNA-LNPs containing sucrose in their core were significantly larger compared to those without internal sucrose, while PDI and EE remained comparable following the preparation process (Fig. 1). After lyophilization under various freezing conditions, trends in particle size, PDI, and EE were consistent with those observed in formulations lacking internal sucrose (Fig. 1). Additionally, mRNA integrity data showed no significant differences between the two formulation, as confirmed by two-way ANOVA (Fig. 2). Interestingly, MFI was significantly higher in both liquid and lyophilized samples of the formulation without sucrose inside the core. This further confirms our data obtained by investigating the different freezing protocols, showing that MFI behaves inversely proportional to particle size (Fig. 3).
  Stability data from vials stored at 2–8°C for six months further supported these finding (Fig. S-2). Over this period, particle size increased and eGFP expression decreased. However, PDI (Fig. S-2A), encapsulated mRNA concentration (Fig. S-2B), and mRNA integrity (Fig. S-2C) remained largely unchanged.
  In summary, the addition of sucrose to the aqueous phase prior to LNP formation provided no measurable benefit.
(D) Modifying mRNA-Lipid Interactions
  Finally, we assessed the effect of incorporating alternative excipients into the inner core of the LNP to modulate the interactions between mRNA and the ionizable lipid. Specifically, 50 mM NaCl or 50 mM L-Methionine were added to the aqueous phase. NaCl led to an increase in particle size in liquid formulation, while L-Methionine resulted in a reduction in EE after lyophilization (Fig. 4). More pronounced differences were observed in eGFP expression: both NaCl- and L-Methionine-containing formulations showed reduced MFI in HeLa cells in the liquid state. After lyophilization, however, eGFP expression levels converged with those of the standard formulation (Fig. 5). At no time point were any characteristics strongly different between the NaCl and L-Methionine formulaion. Furthermore, no differences in mRNA integrity occurred in either the liquid or lyophilized samples (data not shown).

## Discussion

Lyophilization is a promising approach to improve the long-term stability of mRNA-LNPs. However, the process itself introduces various stresses that can affect particle characteristics, product quality, and efficiency. In particular, increases in particle size and reduction in EE are common challenges during mRNA-LNP lyophilization. This study aimed to gain a mechanistic understanding of these effects by investigating the freezing step of the lyophilization process.

We studied the impact of various freezing protocols during lyophilization of mRNA-LNPs, alongside formulation strategies how to best mitigate freezing-induced stresses. Specifically, we compared three different cooling rates (0.1, 0.5, and 1.5 K/min) with a cycle employing controlled nucleation using an ice fog method (CoN). While CoN resulted in improved cake appearance and higher EE, conventional freezing preserved LNP size more effectively, and led to enhanced eGFP expression in HeLa cells. We also explored several formulation strategies to improve LNP stability during freezing. These included: (A) altering local LNP interactions via colloidal crowding, (B) potentially mitigating interfacial stress through the addition of a surfactant, (C) incorporating sucrose within the LNPs to protect mRNA and reduce osmotic pressure, and (D) manipulating interactions between mRNA and the ionizable lipid with formulation components.

### Freezing Protocols

Given that the maximum achievable cooling rates in standard freeze-dryers are typically limited to approximately 2 K/min [36], we investigated rates of 0.1, 0.5, and 1.5 K/min. This range is generally too narrow to significantly affect nucleation temperature, which in our experiments ranged from –13°C to –19°C across all rates. However, cooling rate did influence freezing time, defined as the duration between nucleation and the point when the product temperature realigns with the shelf temperature. More important, the stress time during freezing defined as the time between initial nucleation and solidification of the maximally freeze-concentrated solution was different ranging from minutes to several hours. Previous studies evaluating freezing of monoclonal antibodies or lactate dehydrogenase in histidine buffer have demonstrated that the cooling rate is a primary determinant of cryo-concentration, which can be mitigated by using faster rates [37, 38].

Kasper* et al*. have reported that DNA-polyplexes showed greater size increases following slow freezing ramps after controlled nucleation, likely due to extended residence time in a low-viscosity state before solidification [39].

Transferring this to our nanoparticulate system, although confirmatory studies would be needed, we hypothesize that the increase in particle size observed post-lyophilization may be influenced by cryo-concentration due to prolonged freezing and hence stress times. Faster cooling may reduce the time available for solutes to accumulate in the unfrozen phase, thereby lowering the extent of cryo-concentration. Since conventional freezing conditions exhibited similar degrees of supercooling, they likely induce comparable cryo-concentration effects. This may explain why the fastest rate (1.5 K/min) resulted in the smallest size increase, although the differences were not statistically significant.

As a result, a faster freezing may limit cryo-concentration and thereby reduce particle size increase. Instant freezing using liquid nitrogen has been discussed as an alternative due to its extremely high cooling rate [40]. However, it also produces numerous small ice crystals, which extend primary drying time and significantly increase the ice-liquid interfacial area, and is not preferred in a routine manufacturing setting.

Interfacial stress is another important factor during freezing. In other systems, such as protein formulations, they can adsorb to interfaces, leading to unfolding and denaturation and eventually loss in biological function.

As interfacial stress results from the formation of ice crystals, we hypothesize that the interaction of the LNPs with the newly formed ice-liquid interface may be detrimental to particle integrity resulting in lipid layer destabilization and cargo leakage and hence reduced EE. EE may thus be adversely affected compared to particle size. To strengthen these findings, we evaluated CoN as a method characterized by a low degree of supercooling and the formation of large ice crystals [41]. In general, large ice crystals as a result of slow cooling rates typically facilitate faster primary drying, but require longer secondary drying due to decreased surface area making it more difficult for adsorbed (unfrozen) water to desorb. Consequently, samples subjected to CoN may exhibit higher residual moisture unless secondary drying times are adjusted [16]. In our study, we found that mRNA-LNPs showed higher EE post-lyophilization using CoN but highest increase in particle size compared to other formulations. This further strengthened our hypothesis that reduced interfacial stress as a result of a slower cooling rate and the formation of larger ice crystals contributes to the higher EE observed post-lyophilization, whereas at a longer freezing time, potentially together with the higher (controlled) nucleation temperature at −10°C, allowed for more pronounced cryo-concentration and thus increase in particle size.

Interestingly, despite not modifying secondary drying times, we observed lower residual moisture and superior cake appearance after CoN for our mRNA-LNPs. Micro-CT imaging would be beneficial to evaluate the internal pore structure to elucidate the impact of CoN on moisture levels.

Our *in vitro* experiments in HeLa cells indicated that particle size, not EE, was the primary determinant of reduced MFI, consistent with previous findings. Kong *et al*. demonstrated that smaller LNPs (~ 95 nm) had higher transfection efficiency than medium (~ 120 nm) and large (~ 170 nm) LNPs in HEK293 and DC2.4 cells [42]. Similarly, Yanez Arteta *et al*. showed that protein expression in hepatocytes and adipocytes was highest with 64 nm LNPs, with both larger and smaller sizes performing worse [43]. However, *in vivo* data suggests that size variations may be less critical depending on the species that is studied. For instance, Hassett *et al*. found that while mice required ~ 100 nm LNPs for robust immune responses, non-human primates responded effectively to a broad size range (60–150 nm) [44]. Thus, *in vitro* data may not reliably predict* in vivo *outcomes. Consequently, CoN should continue to be considered in process optimization, even though it showed the poorest results in our cell experiments. However, high EE and superior cake appearance may preferable if particle size is not the most important factor *in vivo.*

### Formulation Strategies

To enhance LNP stability during lyophilization, we investigated the role of colloidal crowding, a strategy previously shown to stabilize mRNA-LNPs during freeze/thaw cycles and lyophilization [26]. In our study, increasing the overall LNP concentration from 0.02 mg/mL to 0.1 mg/mL, using either mRNA-loaded or empty LNPs, resulted in a modest improvement in EE. However, this approach also introduced unintended effects, such as an increase in particle size at higher mRNA-LNP concentration and decreased eGFP expression when empty LNPs were added.

These findings align with observations by Wang *et al*. who reported a sharp decline in EE at 0.1 mg/mL but stable EE at 0.5 mg/mL when studying the effects of the mRNA concentration on lyophilization of mpox mRNA-LNPs. They also noted an increase in particle sizes at higher concentrations [3], consistent with our results.

We hypothesize that reduced interfacial stress as a result of less water per particle, may limit ice crystal formation near LNP surfaces and thus reduce the total ice-liquid interface area.

The stabilization of nanoparticulate systems during lyophilization may be explained by the particle isolation hypothesis, which has been applied to lipid/DNA complexes. According to this hypothesis, spatial separation in the unfrozen fraction prevents particulate aggregation [45]. However, for our high-mRNA-concentration formulation, 10% sucrose may not be sufficient to fully isolate particles, potentially allowing some degree of aggregation or fusion.

Moreover, the addition of empty LNPs reduced both eGFP expression and HeLa cell viability already in liquid state, likely due to increased lipid content relative to mRNA. Although ionizable lipids are generally less cytotoxic than permanently cationic lipids [46], excessive lipid concentrations introduced by empty LNPs appear to have cytotoxic effects.

In sum, our observation of reduced MFI in HeLa cells with lyophilized, high-concentration mRNA-LNPs, despite improved EE, further supports the notion that particle size, rather than EE, is a more critical determinant of *in vitro* transfection efficiency.

As another strategy to mitigate interfacial stress, we added 0.05% Poloxamer 188. In our study, Poloxamer did not adversely affect LNP characteristics but was insufficient to enhance stability during lyophilization. Higher concentrations might be more effective though toxicological limits dependent on the route of administration need to be considered; Li* et al*. for example, used up to 1.5% (w/v) [47] when studying different formulations for thin-film freeze-drying of an influenza A virus hemagglutinin mRNA-LNP vaccine. Another possibility is that Poloxamer as a surfactant integrates into the LNP structure, strongly reducing its concentration at the interface [48, 49]. A patent from Arcturus Therapeutics describes the lyophilization of LNPs using Poloxamer 188 as an excipient. The addition of 0.1–0.2% Poloxamer to 15% cryoprotectant was identified as the optimal condition for small interfering RNA (siRNA)-LNPs with concentrations of 0.5, 1, or 2 mg/mL. However, for 0.25 mg/mL siRNA-LNPs, a formulation without Poloxamer was found to be more favorable. Furthermore, it was demonstrated that adding Poloxamer 188 after lyophilization as part of reconstitution was determined to best maintain EE. As illustrated, siRNA-LNPs are employed in the aforementioned example; however, in certain embodiments, the RNA may be mRNA or self-replicating RNA, with a maximum length of 13,000 nucleotides [50]. Consequently, higher concentrations of Poloxamer 188 might be needed to show beneficial effects in our experiments. Especially higher concentrations of Poloxamer 188 at higher mRNA–LNP concentrations could be beneficial, as in our experiments higher concentrations of mRNA–LNPs better preserved EE but led to an increase in particle size, which may be mitigated through the addition of a surfactant. To note, adding a surfactant to the liquid formulation may help mitigate the formation of white deposits, presumably LNP components, on the surfaces of vials caused by shaking [25].

We further tested how reduced osmotic stress may influence EE. In liposome studies, protectants were added both inside and outside liposomes to balance osmotic pressure [11, 51, 52]. To investigate this in our system, we formulated LNPs with sucrose incorporated in the core of the LNP. Additionally, we hypothesized that sucrose might stabilize the mRNA by replacing hydrogen bonds during potential removal of water during drying. However, no improvement in EE, nor in mRNA integrity and eGFP expression in HeLa cells, was observed, suggesting that other stress factors, e.g., interfacial stress, rather than osmotic imbalance, may the primary cause of EE reduction during lyophilization.

Finally, we evaluated the effects of L-Methionine and NaCl on the interactions between mRNA and ionizable lipids. Due to its zwitterionic nature and antioxidant properties, we hypothesized that L-Methionine may strengthen interactions and stability, while the increased ionic strength through NaCl may weaken electrostatic interactions. However, neither excipient improved stability nor majorly destabilized the LNP system. In fact, both excipients increased particle size and led to reduced cellular activity, consistent with our previous findings. There was also no influence on mRNA integrity with the addition of L-Methionine or NaCl. The results regarding the addition of NaCl are consistent with findings of Shirane *et al*. who developed an “alcohol dilution-lyophilization method” for mRNA-LNPs and investigated different pH values (pH 3–5) and NaCl buffer concentrations (0 −750 mg/mL). 50 mM NaCl corresponds to 3 mg/mL. At pH 4, only NaCl concentrations higher than 150 mg/mL negatively impact PDI, and 750 mg/mL of NaCl is needed to reduce EE [20].

To note, the use of polyA as a surrogate may sometimes be beneficial from a technical development perspective. However, the present study also shows that e.g., the size increase and changes in EE after CoN as shown for EGFP mRNA-LNPs were not fully reflected by polyA-LNPs. In contrast, changes in polyA-LNP characteristics may translate into eGFP mRNA-LNP characteristics representing a best case scenario.

To conclude, our results show that increases in particle size and reductions in EE often follow opposing trends across freezing conditions. Therefore, the optimal freezing protocol must be tailored to each specific formulation and lipid composition to ensure both physical stability and biological efficacy.

## Conclusions

A deeper understanding of the freezing process during lyophilization and the impact of freezing-induced stresses on mRNA-LNP stability is essential for the rational design of both formulation and process parameters for lyophilized mRNA-LNPs. Our results show that a decrease in EE was more pronounced at faster cooling rates, while at slower cooling rates, the increases in particle size was largest. Based on these findings, we hypothesize that the increase in size of LNPs post-lyophilization may be primarily driven by cryo-concentration as a result of longer freezing times, whereas losses in EE may be influenced by ice–liquid interfacial stress as a result of smaller ice crystals formed during faster freezing protocols. To our knowledge, this is the first study to provide systematic experimental evidence of the impact of freezing conditions during lyophilization on mRNA–LNPs. Our results demonstrate that, due to opposing trends in particle size increase and mRNA leakage, process optimization alone cannot fully stabilize mRNA–LNPs, and an improved formulation is therefore required.

The addition of 0.05% Poloxamer 188 was insufficient to mitigate interfacial stress. While colloidal crowding slightly improved EE, it introduced drawbacks such as increased particle size at high mRNA-LNP concentrations and reduced eGFP expression when empty LNPs were added. Unlike with liposomes, incorporation of sucrose into the LNP core did not reduce leakage, supporting the notion that interfacial stress rather than osmotic pressure drives reduced EE. Moreover, inclusion of sodium chloride or L-Methionine into the LNP core did not modify final LNP stability.

Although the tested formulation strategies did not successfully reduce lyophilization-induced stress, especially our findings regarding freezing protocols enhance the mechanistic understanding of stress factors during freezing of the lyophilization process of mRNA-LNPs. This knowledge provides a foundation for future optimization of mRNA-LNP formulations and their freezing protocols during freeze-drying.

## Supplementary Information

Below is the link to the electronic supplementary material.

ESM 1 (DOCX 609 MB)

## Data Availability

Data will be made available on request.

## References

[1] Muramatsu H, Lam K, Bajusz C, Laczkó D, Karikó K, Schreiner P *et al.* Lyophilization provides long-term stability for a lipid nanoparticle-formulated nucleoside-modified mRNA vaccine. Mol Ther. 2022. 10.1016/j.ymthe.2022.02.001. 10.1016/j.ymthe.2022.02.001 PMC8815268 35131437

[2] Ai L, Li Y, Zhou L, Yao W, Zhang H, Hu Z, *et al*. Lyophilized mRNA-lipid nanoparticle vaccines with long-term stability and high antigenicity against SARS-CoV-2. Cell Discov. 2023;9(1):9. 36683074 10.1038/s41421-022-00517-9 PMC9868121

[3] Wang B, Yin Q, Yi L, Su C, Wen Y, Qiao M, *et al*. Lyophilized monkeypox mRNA lipid nanoparticle vaccines with long-term stability and robust immune responses in mice. Hum Vaccin Immunother. 2025;21(1):2477384. 40066621 10.1080/21645515.2025.2477384 PMC11901372

[4] Lamoot A, Lammens J, Lombaerde E, Zhong Z, Gontsarik M, Chen Y *et al*. Successful batch and continuous lyophilization of mRNA LNP formulations depend on cryoprotectants and ionizable lipids. Biomater Sci. 2023. 10.1039/d2bm02031a 10.1039/d2bm02031a 37073472

[5] Meulewaeter S, Nuytten G, Cheng MHY, Smedt SC, Cullis PR, Beer T, *et al*. Continuous freeze-drying of messenger RNA lipid nanoparticles enables storage at higher temperatures. J Control Release. 2023;357:149–60. 36958400 10.1016/j.jconrel.2023.03.039 PMC10062427

[6] Ruppl A, Kiesewetter D, Köll-Weber M, Lemazurier T, Süss R, Allmendinger A. Formulation screening of lyophilized mRNA-lipid nanoparticles. Int J Pharm. 2025;671: 125272. 39875036 10.1016/j.ijpharm.2025.125272

[7] Fan Y. Physicochemical and structural insights into lyophilized mRNA-LNP from lyoprotectant and buffer screenings. J Contr Release. 2024. 10.1016/j.jconrel.2024.07.052. 10.1016/j.jconrel.2024.07.052 39059500

[8] Zhao P, Hou X, Yan J, Du S, Xue Y, Li W, *et al*. Long-term storage of lipid-like nanoparticles for mRNA delivery. Bioact Mater. 2020;5(2):358–63. 32206737 10.1016/j.bioactmat.2020.03.001 PMC7078456

[9] Li M, Jia L, Xie Y, Ma W, Yan Z, Liu F, *et al*. Lyophilization process optimization and molecular dynamics simulation of mRNA-LNPs for SARS-CoV-2 vaccine. NPJ Vaccines. 2023;8(1):153. 37813912 10.1038/s41541-023-00732-9 PMC10562438

[10] Crowe J, Crowe LM, Carpenter JF. Preserving dry biomaterials: The water replacement hypothesis. BioPharm. 1993;6:28–37.

[11] Franzé S, Selmin F, Samaritani E, Minghetti P, Cilurzo F. Lyophilization of liposomal formulations: still necessary, still challenging. pharmaceutics. 2018;10(3). 10.3390/pharmaceutics10030139. 10.3390/pharmaceutics10030139 PMC6161153 30154315

[12] Koster KL, Webb MS, Bryant G, Lynch DV. Interactions between soluble sugars and POPC (1-palmitoyl-2-oleoylphosphatidylcholine) during dehydration: vitrification of sugars alters the phase behavior of the phospholipid. Biochimica et Biophysica Acta (BBA) - Biomembranes. 1994;1193(1):143–50. 8038184 10.1016/0005-2736(94)90343-3

[13] Kristiansen J. Leakage of a trapped fluorescent marker from liposomes: effects of eutectic crystallization of NaCl and internal freezing. Cryobiology. 1992;29(5):575–84. 1424714 10.1016/0011-2240(92)90062-7

[14] Trenkenschuh E, Friess W. Freeze-drying of nanoparticles: How to overcome colloidal instability by formulation and process optimization. Eur J Pharm Biopharm. 2021;165:345–60. 34052428 10.1016/j.ejpb.2021.05.024

[15] Chang BS, Kendrick BS, Carpenter JF. Surface-Induced Denaturation of Proteins during Freezing and its Inhibition by Surfactants. J Pharm Sci. 1996;85(12):1325–30. 8961147 10.1021/js960080y

[16] Kasper JC, Friess W. The freezing step in lyophilization: physico-chemical fundamentals, freezing methods and consequences on process performance and quality attributes of biopharmaceuticals. Eur J Pharm Biopharm. 2011;78(2):248–63. 21426937 10.1016/j.ejpb.2011.03.010

[17] Thorat AA, Suryanarayanan R. Characterization of Phosphate Buffered Saline (PBS) in Frozen State and after Freeze-Drying. Pharm Res. 2019;36(7):98. 31087169 10.1007/s11095-019-2619-2

[18] Heller MC, Carpenter JF, Randolph TW. Manipulation of Lyophilization-Induced Phase Separation: Implications For Pharmaceutical Proteins. Biotechnol Prog. 1997;13(5):590–6. 9336978 10.1021/bp970081b

[19] Randolph TW. Phase separation of excipients during lyophilization: effects on protein stability. J Pharm Sci. 1997;86(11):1198–203. 9383725 10.1021/js970135b

[20] Shirane D, Tanaka H, Sakurai Y, Taneichi S, Nakai Y, Tange K, *et al*. Development of an Alcohol Dilution-Lyophilization Method for the Preparation of mRNA-LNPs with Improved Storage Stability. Pharmaceutics. 2023;15(7):1819. 37514007 10.3390/pharmaceutics15071819 PMC10383539

[21] Goldman J, Hassett K, Peng X, Sullivan S. Lyophilization Methods for Preparing Lipid Formulated Ther- apeutics”. U.S. pat. 2024/0226028 A1. ModernaTX, Inc. 2024.

[22] Suzuki Y, Miyazaki T, Muto H, Kubara K, Mukai Y, Watari R, *et al*. Design and lyophilization of lipid nanoparticles for mRNA vaccine and its robust immune response in mice and nonhuman primates. Mol Ther Nucleic Acids. 2022;30:226–40. 36187052 10.1016/j.omtn.2022.09.017 PMC9508692

[23] Chen H, Ren X, Xu S, Zhang D, Han T. Optimization of lipid nanoformulations for effective mRNA delivery. Int J Nanomedicine. 2022;17:2893–905. 35814615 10.2147/IJN.S363990 PMC9259059

[24] Bender V, Fuchs L, Süss R. RP-HPLC-CAD method for the rapid analysis of lipids used in lipid nanoparticles derived from dual centrifugation. International Journal of Pharmaceutics: X. 2024;7: 100255. 38766478 10.1016/j.ijpx.2024.100255 PMC11101883

[25] Ruppl A, Kiesewetter D, Strütt F, Köll-Weber M, Süss R, Allmendinger A. Don’t shake it! Mechanical stress testing of mRNA-lipid nanoparticles. Eur J Pharm Biopharm. 2024;198: 114265. 38492867 10.1016/j.ejpb.2024.114265

[26] Darvari R. “What Can Be Learned from Blank Lipid Nanoparticles: A Closer Look”. 14th European and Global Summit for Clinical Nanomedicine (Basel). 2023.

[27] Center for Drug Evaluation and Research. FDA; 2025 [accessed 2025 May 15]. Inactive Ingredients Database Download. Available from: https://www.fda.gov/drugs/drug-approvals-and-databases/inactive-ingredients-database-download. Accessed 15 May 2025.

[28] McShan AC, Kei P, Ji JA, Kim DC, Wang YJ. Hydrolysis of polysorbate 20 and 80 by a range of carboxylester hydrolases. PDA J Pharm Sci Technol. 2016;70(4):332–45. 27020650 10.5731/pdajpst.2015.005942

[29] Yao J, Dokuru DK, Noestheden M, Park SS, Kerwin BA, Jona J, *et al*. A quantitative kinetic study of polysorbate autoxidation: the role of unsaturated fatty acid ester substituents. Pharm Res. 2009;26(10):2303–13. 19669100 10.1007/s11095-009-9946-7

[30] Zhang L, Yadav S, Demeule B, Wang YJ, Mozziconacci O, Schӧneich C. Degradation mechanisms of polysorbate 20 differentiated by ^18^O-labeling and mass spectrometry. Pharm Res. 2017;34(1):84–100. 27738952 10.1007/s11095-016-2041-y

[31] Bollenbach L, Buske J, Mäder K, Garidel P. Poloxamer 188 as surfactant in biological formulations – an alternative for polysorbate 20/80? Int J Pharm. 2022;620: 121706. 35367584 10.1016/j.ijpharm.2022.121706

[32] Rieser R, Menzen T, Biel M, Michalakis S, Winter G. Systematic studies on stabilization of AAV vector formulations by lyophilization. J Pharm Sci. 2022;111(8):2288–98. 35259349 10.1016/j.xphs.2022.03.004

[33] Li J, Sonje J, Suryanarayanan R. Role of poloxamer 188 in preventing ice-surface-induced protein destabilization during freeze-thawing. Mol Pharmaceutics. 2023;20(9):4587–96. 10.1021/acs.molpharmaceut.3c00312 37535010

[34] Trenkenschuh E, Richter M, Heinrich E, Koch M, Fuhrmann G, Friess W. Enhancing the stabilization potential of lyophilization for extracellular vesicles. Adv Healthc Mater. 2022;11(5): 2100538. 34310074 10.1002/adhm.202100538 PMC11468620

[35] Soeda K, Arai K, Yamamoto T, Ofuji K, Fukuda M, Hashimoto D, *et al*. Mechanism of protein-PDMS visible particles formation in liquid vial monoclonal antibody formulation. J Pharm Sci. 2023;112(3):653–64. 36191621 10.1016/j.xphs.2022.09.027

[36] Tang X, Pikal MJ. Design of freeze-drying processes for pharmaceuticals: practical advice. Pharm Res. 2004;21(2):191–200. 15032301 10.1023/b:pham.0000016234.73023.75

[37] Peláez SS, Mahler HC, Huwyler J, Allmendinger A. Directional freezing and thawing of biologics in drug substance bottles. Eur J Pharm Biopharm. 2024;203: 114427. 39094667 10.1016/j.ejpb.2024.114427

[38] Minatovicz B, Sansare S, Mehta T, Bogner RH, Chaudhuri B. Large-scale freeze-thaw of protein solutions: study of the relative contributions of freeze-concentration and ice surface area on stability of lactate dehydrogenase. J Pharm Sci. 2023;112(2):482–91. 36162492 10.1016/j.xphs.2022.09.020

[39] Kasper JC, Pikal MJ, Friess W. Investigations on polyplex stability during the freezing step of lyophilization using controlled ice nucleation–the importance of residence time in the low-viscosity fluid state. J Pharm Sci. 2013;102(3):929–46. 23280536 10.1002/jps.23419

[40] Heller MC, Carpenter JF, Randolph TW. Protein formulation and lyophilization cycle design: prevention of damage due to freeze-concentration induced phase separation. Biotechnol Bioeng. 1999;63(2):166–74. 10099593 10.1002/(sici)1097-0290(19990420)63:2<166::aid-bit5>3.0.co;2-h

[41] Rambhatla S, Ramot R, Bhugra C, Pikal MJ. Heat and mass transfer scale-up issues during freeze drying: II. Control and characterization of the degree of supercooling. AAPS PharmSciTech. 2004;5(4):54–62. 10.1208/pt050458 PMC2750483 15760055

[42] Kong W, Wei Y, Dong Z, Liu W, Zhao J, Huang Y, *et al*. Role of size, surface charge, and PEGylated lipids of lipid nanoparticles (LNPs) on intramuscular delivery of mRNA. Journal of Nanobiotechnology. 2024;22(1):553. 39261807 10.1186/s12951-024-02812-x PMC11389890

[43] Yanez Arteta M, Kjellman T, Bartesaghi S, Wallin S, Wu X, Kvist AJ, *et al*. Successful reprogramming of cellular protein production through mRNA delivered by functionalized lipid nanoparticles. Proc Natl Acad Sci. 2018;115(15):E3351–60. 29588418 10.1073/pnas.1720542115 PMC5899464

[44] Hassett KJ, Higgins J, Woods A, Levy B, Xia Y, Hsiao CJ, *et al*. Impact of lipid nanoparticle size on mRNA vaccine immunogenicity. J Control Release. 2021;335:237–46. 34019945 10.1016/j.jconrel.2021.05.021

[45] Allison SD, Molina MdC, Anchordoquy TJ. Stabilization of lipid/DNA complexes during the freezing step of the lyophilization process: the particle isolation hypothesis. Biochimica et Biophysica Acta (BBA) - Biomembranes. 2000;1468(1–2):127–38. 11018658 10.1016/s0005-2736(00)00251-0

[46] Sun D, Lu ZR. Structure and function of cationic and ionizable lipids for nucleic acid delivery. Pharm Res. 2023;40(1):27–46. 36600047 10.1007/s11095-022-03460-2 PMC9812548

[47] Li Q, Shi R, Xu H, AboulFotouh K, Sung MMH, Oguin TH, *et al*. Thin-film freeze-drying of an influenza virus hemagglutinin mRNA vaccine in unilamellar lipid nanoparticles with blebs. J Control Release. 2024;375:829–38. 39293526 10.1016/j.jconrel.2024.09.030

[48] Heerklotz H. Interactions of surfactants with lipid membranes. Q Rev Biophys. 2008;41(3–4):205–64. 19079805 10.1017/S0033583508004721

[49] Wu G, Lee KYC. Interaction of poloxamers with liposomes: an isothermal titration calorimetry study. J Phys Chem B. 2009;113(47):15522–31. 19863124 10.1021/jp906331m

[50] Sagi A, Bao Y, Karmali P Prakash. “Method of Lyophilizing Lipid Nanoparticles”. Pat. WO 2022/036170 A1. Arcturus Therapeutics.

[51] Kannan V, Balabathula P, Thoma LA, Wood GC. Effect of sucrose as a lyoprotectant on the integrity of paclitaxel-loaded liposomes during lyophilization. J Liposome Res. 2015;25(4):270–8. 25534990 10.3109/08982104.2014.992023

[52] Crowe JH, Crowe LM. Factors affecting the stability of dry liposomes. Biochimica et Biophysica Acta (BBA) - Biomembranes. 1988;939(2):327–34. 3355821 10.1016/0005-2736(88)90077-6

