# Supplementary material for: Freezing-Induced Stress in mRNA-Lipid Nanoparticles During Lyophilization: Mechanistic Insights From Process and Formulation Studies
Source: Pharm Res. 2026 Feb 20;43(3):877–89. doi: 10.1007/s11095-026-04039-x (PMC13076365; doi:10.1007/s11095-026-04039-x)
Supplement: Supplementary file 1 — (DOCX 609 MB) [file 11095_2026_4039_MOESM1_ESM.docx]

# Supporting Information

**Tab. S-1: Exemplary pictures of vials after lyophilization with a cooling rate of 0.1 K/min, 0.5 K/min, or 1.5 K/min or using CoN. For mRNA-LNPs formulated with 10% (w/v) sucrose outside (mRNA), for mRNA-LNPs formulated with 10% (w/v) sucrose inside and outside (mRNA Suc), and for polyA-LNPs formulated with 10% (w/v) sucrose outside (polyA)**

|  | 0.1 K/min | 0.5 K/min | 1.5 K/min | CoN |
| --- | --- | --- | --- | --- |
| mRNA |  |  |  |  |
| mRNA Suc |  |  |  |  |
| polyA |  |  |  |  |

**Fig. S-1: Residual moisture of polyA-LNPs after lyophilization with a cooling rate of 0.1 K/min, 0.5 K/min, or 1.5 K/min or using CoN. Statistical analysis was performed using a one-way ANOVA with Tukey’s multiple comparisons test.**

| A   | B   |
| --- | --- |
| C   | D   |

**Fig. S-2: Stability data after 6 months storage at 5° C of mRNA-LNPs in liquid and lyophilized dosage form. Lyophilized formulations applied a freezing protocol at 1.5 K/min. LNPs were analyzed for (A) size and PDI, (B) the encapsulated mRNA concentration, (C) mRNA integrity, and (D) eGFP-expression in HeLa cells. Data before storage are the same as shown in Fig. 1+2. Following number of vials were stored at 5 °C: 1 vial mRNA-LNPs liquid, 3 vials mRNA-LNPs lyo, and 2 vials mRNA-LNPs Suc lyo. Statistical analysis was performed using a two-way ANOVA with Tukey’s multiple comparisons test to analyze variations between before and after storage as well as adding sucrose before mixing of the lyophilized samples.**

**Fig. S-3: Exemplary electropherogram of extracted eGFP mRNA after lyophilization. Area of the low molecular weight species and area of the main species (in red) were integrated. Percentage area of main peak was used to assess mRNA integrity.**
